# Vertebral bone marrow fat fraction changes in postmenopausal women with breast cancer receiving combined aromatase inhibitor and bisphosphonate therapy

**DOI:** 10.1186/s12891-019-2916-2

**Published:** 2019-11-06

**Authors:** Michael Dieckmeyer, Stefan Ruschke, Alexander Rohrmeier, Jan Syväri, Ingo Einspieler, Vanadin Seifert-Klauss, Monika Schmidmayr, Stephan Metz, Jan S. Kirschke, Ernst J. Rummeny, Claus Zimmer, Dimitrios C. Karampinos, Thomas Baum

**Affiliations:** 1 Department of Diagnostic and Interventional Neuroadiology, Klinikum rechts der Isar, Technische Universität München, Munich, Germany; 2 Department of Radiology, Klinikum rechts der Isar, Technische Universität München, Munich, Germany; 3 Department of Radiology, University Medical Center Regensburg, Regensburg, Germany; 4 Department of Gynecology and Obstetrics, Klinikum rechts der Isar, Technische Universität München, Munich, Germany

**Keywords:** Osteoporosis, Bisphosphonates, Aromatase inhibitor, Vertebral bone marrow, Proton density fat fraction

## Abstract

**Background:**

Quantification of vertebral bone marrow (VBM) water–fat composition has been proposed as advanced imaging biomarker for osteoporosis. Estrogen deficiency is the primary reason for trabecular bone loss in postmenopausal women. By reducing estrogen levels aromatase inhibitors (AI) as part of breast cancer therapy promote bone loss. Bisphosphonates (BP) are recommended to counteract this adverse drug effect. The purpose of our study was to quantify VBM proton density fat fraction (PDFF) changes at the lumbar spine using chemical shift encoding-based water-fat MRI (CSE-MRI) and bone mineral density (BMD) changes using dual energy X-ray absorptiometry (DXA) related to AI and BP treatment over a 12-month period.

**Methods:**

Twenty seven postmenopausal breast cancer patients receiving AI therapy were recruited for this study. 22 subjects completed the 12-month study. 14 subjects received AI and BP (AI+BP), 8 subjects received AI without BP (AI-BP).

All subjects underwent 3 T MRI. An eight-echo 3D spoiled gradient-echo sequence was used for CSE-based water-fat separation at the lumbar spine to generate PDFF maps. After manual segmentation of the vertebral bodies L1-L5 PDFF values were extracted for each vertebra and averaged for each subject.

All subjects underwent DXA of the lumbar spine measuring the average BMD of L1-L4.

**Results:**

Baseline age, PDFF and BMD showed no significant difference between the two groups (*p* > 0.05). There was a relative longitudinal increase in mean PDFF (∆rel_PDFF_) in both groups (AI+BP: 5.93%; AI-BP: 3.11%) which was only significant (*p* = 0.006) in the AI+BP group. ∆rel_PDFF_ showed no significant difference between the two groups (*p* > 0.05). There was no significant longitudinal change in BMD (p > 0.05).

**Conclusions:**

Over a 12-month period, VBM PDFF assessed with CSE-MRI significantly increased in subjects receiving AI and BP. The present results contradict previous results regarding the effect of only BP therapy on bone marrow fat content quantified by magnetic resonance spectroscopy and bone biopsies. Future longer-term follow-up studies are needed to further characterize the effects of combined AI and BP therapy.

## Background

Vertebral bone marrow fat fraction (BMFF) has been shown to be related to age, anatomical location, hormone levels as well as a variety of medical conditions or treatments, such as osteoporosis [1–6], diabetes [4, 7, 8], radiation therapy and chemotherapy [9, 10]. In the context of osteoporosis, it has been established that bone loss is associated with an increase in vertebral BMFF [11–13]. The altered differentiation of mesenchymal progenitor cells is one of several mechanisms on the cellular level causing this increase. These cells can differentiate into osteoblasts and osteocytes or into adipocytes. It was shown that with aging there is a shift to a more adipogenic fate [14].

Chemical shift encoded magnetic resonance imaging (CSE-MRI) and magnetic resonance spectroscopy (MRS) constitute two techniques that enable non-invasive in-vivo measurement of vertebral BMFF [15]. Previous studies have shown that proton density fat fraction (PDFF) is the parameter of choice when it comes to the assessment of vertebral bone marrow water-fat composition [16, 17].

Aromatase inhibitor (AI) therapy is a standard treatment component for estrogen receptor positive breast cancer in postmenopausal women [18–20]. By reducing estrogen levels, it inhibits tumor cell growth. However, reduced estrogen levels also promote bone loss and the development of osteoporosis. To counteract this adverse drug effect, bisphosphonates (BP) can be administered which are recommended as first line antiosteoporotic therapy [21, 22] and have been shown to increase bone mineral density (BMD) [23], reduce fracture risk [24] and reduce BMFF [25] in postmenopausal osteoporotic women. The current clinical gold standard to assess osteoporosis-associated fracture risk in these patients is the determination of BMD using dual-energy X-ray absorptiometry (DXA). However, DXA-based BMD values of subjects with and without osteoporotic fractures overlap [26]. Therefore, advanced imaging biomarkers are needed to improve the prediction of fracture risk beyond BMD.

The purpose of the present study was to quantify changes in vertebral BMFF and BMD over one year in postmenopausal breast cancer patients receiving AI therapy with and without additional BP therapy, respectively. We hypothesized that vertebral BMFF measurements are more sensitive to medication induced changes than DXA-based BMD measurements.

## Methods

### Subjects

For this study 27 postmenopausal female breast cancer patients receiving AI therapy were recruited at the Department of Gynecology, Klinikum rechts der Isar, Technical University of Munich, Germany. The time between breast cancer diagnosis and start of AI therapy ranged between three and five months. Exclusion criteria were past or current chemotherapy, history of bone metastasis or vertebral fractures, past or current intake of medication affecting bone metabolism (other than calcium and vitamin D), history of hemato-oncological disease, impaired renal function as well as general MRI contraindications. Five subjects dropped out due to disease progression during follow-up resulting in a total of 22 subjects completing the 12-month follow-up study (baseline: age = 62.3 ± 6.5 years, BMI = 25.4 ± 4.2 kg/m^2^; follow-up: age = 63.3 ± 7.2 years, BMI = 25.6 ± 4.2 kg/m^2^). Based on the multidisciplinary tumor board recommendations, 14 subjects received AI and BP (zoledronic acid) therapy (AI+BP) and 8 subjects received AI without BP therapy (AI-BP).

### Magnetic resonance imaging

All subjects underwent 3 T MRI (Ingenia, Philips Healthcare, Best, Netherlands). An eight-echo 3D spoiled gradient-echo sequence was used for CSE-based water-fat separation at the lumbar spine using the built-in-the-table posterior coil elements (12-channel array). 8 echoes were acquired in a single TR using non-flyback (bipolar) read-out gradients and the following imaging parameters: TR/TE1/ΔTE = 11/1.4/1.1 ms, FOV = 220 × 220 × 80 mm^3^, acquisition matrix = 124 × 121, voxel size = 1.8 × 1.8 × 4.0 mm^3^, receiver bandwidth = 1527 Hz/pixel, frequency direction = A/P (to minimize breathing artifacts), 1 average, scan time = 1 min 17 s. A flip angle of 3° was used to minimize T1-bias effects.

**Fig. 1:**
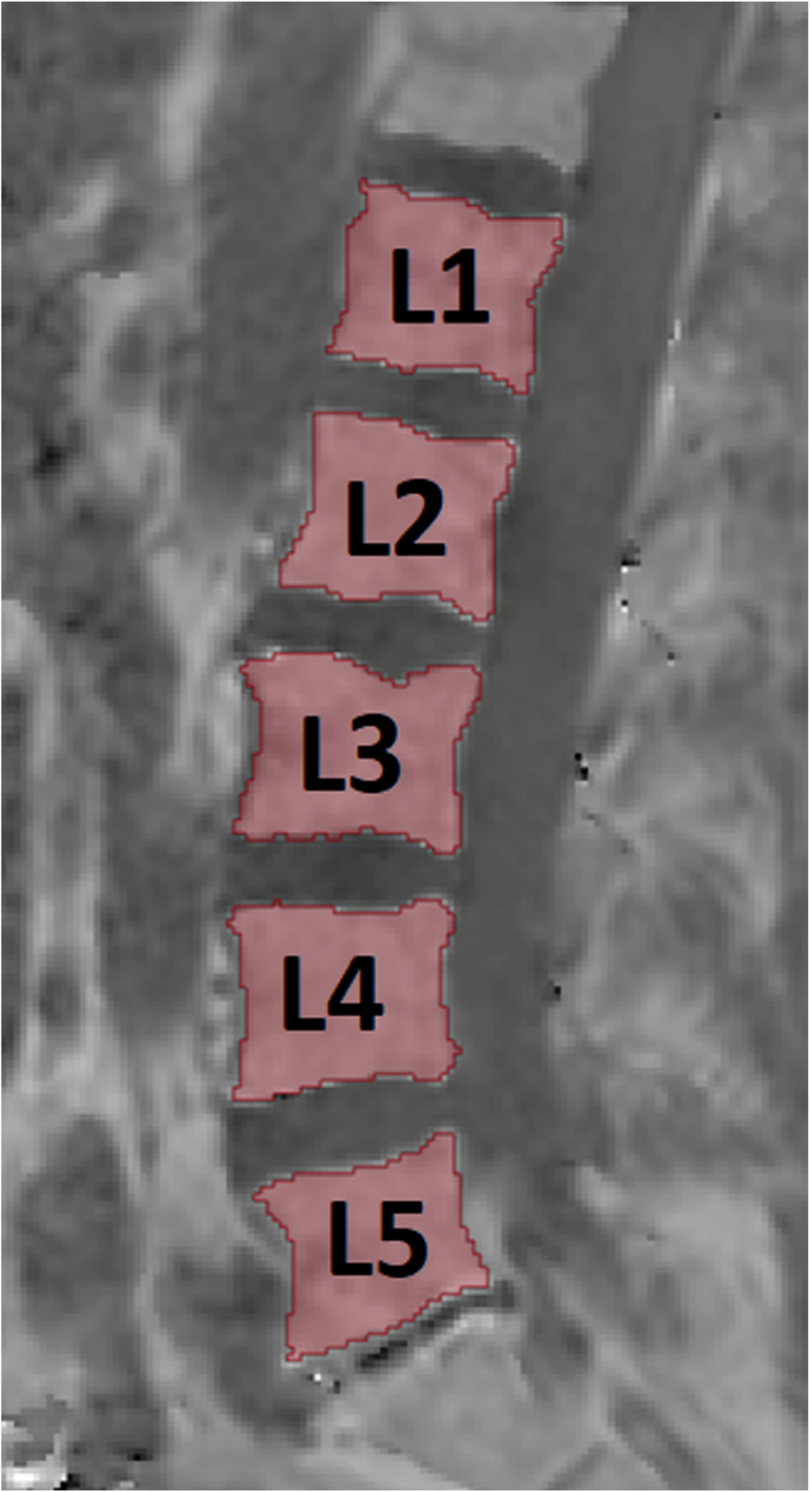
Manually segmented regions of interest (ROIs) in the L1-L5 vertebral bodies (red), drawn on the calculated PDFF map of the eight-echo 3D spoiled gradient-echo sequence using MITK

The gradient echo imaging data was processed online using the fat quantification routine of the vendor. The routine first performs a phase error correction and then a complex-based water–fat decomposition using a pre-calibrated seven-peak fat spectrum and a single T2* to model the signal variation with echo time and compute PDFF maps. Segmentation of the vertebral bodies L1 to L5 was performed manually by a radiologist on the PDFF maps using the open-source software Medical Imaging Interaction Toolkit (MITK) (Fig. 1). Vertebrae with degenerative changes, e.g. Modic changes, and benign lesions, e.g. hemangiomas, were excluded. PDFF values were extracted at each vertebral level from L1 to L5 and averaged for each subject. Reproducibility error values of vertebral PDFF measurements at the lumbar spine were reported previously [27] and amounted to 1.7% (absolute unit) over C3 to L5. The relative longitudinal change in PDFF (∆rel _PDFF_) was defined as.

$$ {\Delta \mathrm{rel}}_{\mathrm{PDFF}}=\left({\mathrm{PDFF}}_{\mathrm{follow}-\mathrm{up}}-{\mathrm{PDFF}}_{\mathrm{baseline}}\right)/{\mathrm{PDFF}}_{\mathrm{baseline}} $$

### BMD measurements

All subjects underwent a medically indicated DXA scan (Lunar Prodigy, GE Healthcare) of the lumbar spine measuring the average areal BMD of L1 to L4. The relative longitudinal change in BMD (∆rel _BMD_) was defined as.

$$ {\Delta \mathrm{rel}}_{\mathrm{BMD}}=\left({\mathrm{BMD}}_{\mathrm{follow}-\mathrm{up}}-{\mathrm{BMD}}_{\mathrm{baseline}}\right)/{\mathrm{BMD}}_{\mathrm{baseline}} $$

### Statistical analysis

All statistical analyses were performed using MATLAB (The MathWorks Inc., Natick, MA, USA) and SPSS (SPSS Inc., Chicago, IL, USA). The Kolmogorov–Smirnov test indicated normally distributed data for the majority of parameters. Differences in the measured variables and relative longitudinal changes between the two treatment groups (AI+BP vs. AI-BP) were tested for significance using unpaired t-tests. Differences in the measured variables between baseline and follow-up measurements in each group were tested for significance using paired t-tests. Statistical tests were performed using a two-sided level of significance α = 0.05.

## Results

Baseline age, BMI, BMD, and PDFF showed no significant (*p* > 0.05) difference between the AI+BP and AI-BP group. Similarly, these parameters were not significantly different between the two treatment groups at 12-month follow-up (Tab. 1 and 2, p > 0.05).

**Table 1:** Anthropometric data (mean ± standard deviation) and *p*-values for unpaired t-tests between the two treatment groups

	Baseline			Follow-up		
	AI+BP (*n* = 14)	AI-BP (*n* = 8)	*p*-value	AI+BP (*n* = 14)	AI-BP (*n* = 8)	*p*-value
Age [y]	61.2 ± 5.5	64.2 ± 8.2	0.314	62.2 ± 5.3	65.2 ± 8.2	0.303
BMI [kg/m^2^]	25.1 ± 4.3	25.9 ± 4.1	0.657	25.5 ± 4.3	25.9 ± 4.1	0.842

**Table 2:** Measured data (mean ± standard deviation) and *p*-values for unpaired t-tests between the two treatment groups

	Baseline			Follow-up		
	AI+BP (*n* = 14)	AI-BP (*n* = 8)	*p*-value	AI+BP (*n* = 14)	AI-BP (*n* = 8)	*p*-value
PDFF [%]	45.66 ± 9.72	45.88 ± 6.95	0.956	48.40 ± 10.69	47.03 ± 7.25	0.750
BMD [g/cm^2^]	1.247 ± 0.252	1.186 ± 0.179	0.554	1.270 ± 0.270	1.177 ± 0.171	0.390

There was a positive ∆rel_PDFF_ averaged over L1 to L5 in both groups (AI+BP: 5.93%; AI-BP: 3.11%), however it was only significant (*p* = 0.006) in the AI+BP group. ∆rel_PDFF_ showed no significant difference between the two groups. There was no significant longitudinal change in BMD (Tab. 3). There was no significant (*p* > 0.05) correlation between PDFF and BMD for the entire patient group or any of the two treatment groups at baseline or follow-up.

**Table 3:** Relative longitudinal change in measured data (mean). *p*-values refer to (un-)paired t-tests

Longitudinal change	All subjects (*n* = 22)	AI+BP (*n* = 14)	AI-BP (*n* = 8)	*p*-value (AI+BP vs. AI-BP)
∆rel_PDFF_ [%]	4.90 (*p* = 0.022)	5.93 (*p* = 0.006)	3.11 (*p* = 0.52)	0.510
∆rel_BMD_ [%]	0.96 (*p* = 0.288)	1.81 (*p* = 0.056)	−0.51 (*p* = 0.792)	0.214

**Fig. 2:**
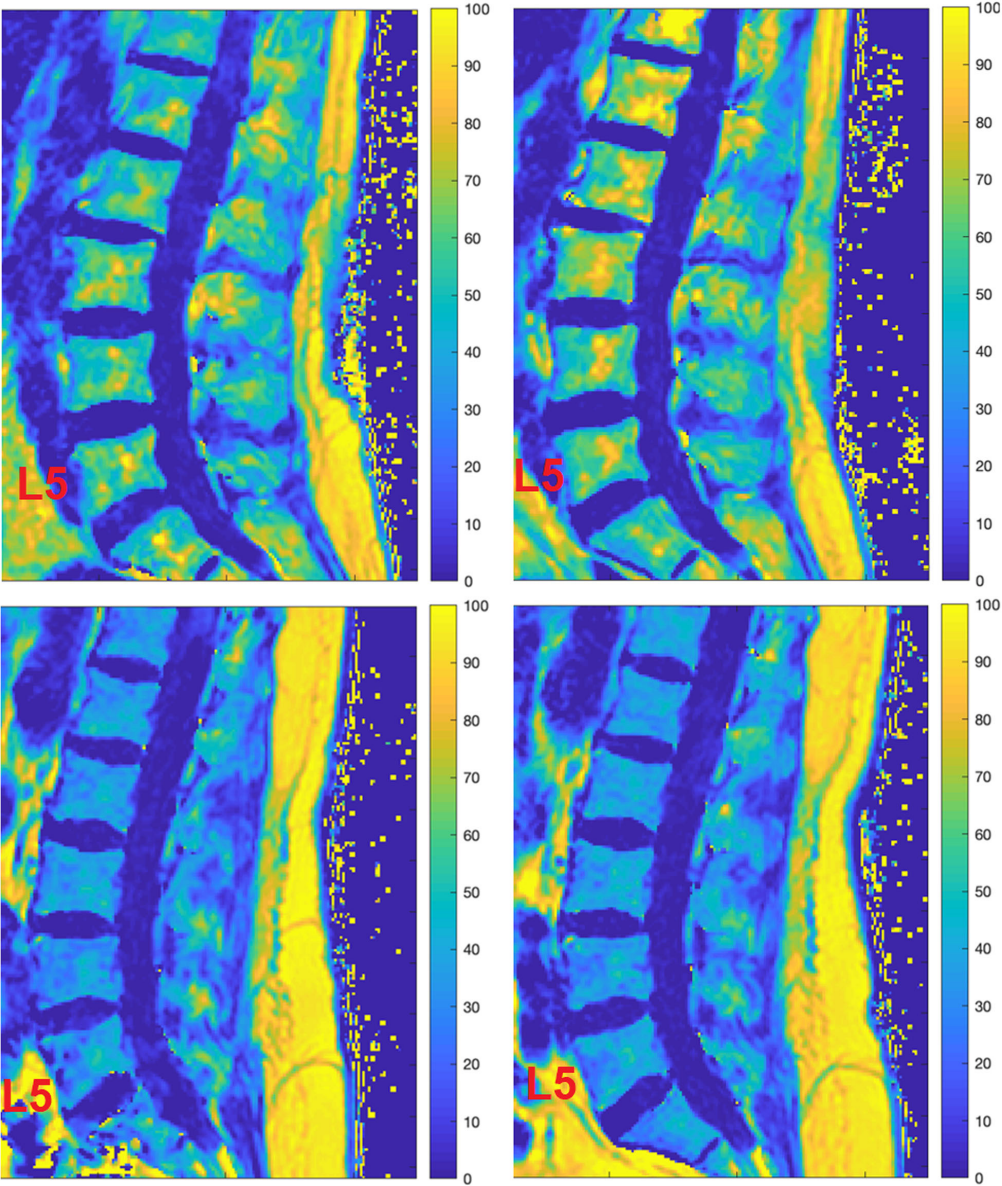
PDFF maps [%] of a patient from the AI+BP group (upper row) and the AI-BP group (bottom row), respectively, at baseline (left column) and follow-up (right column). Mean PDFF values averaged over L1 to L5 at baseline and follow-up were equal to 50.16 and 55.80%, respectively, for the AI+BP patient (upper row); and equal to 30.78 and 34.14%, respectively, for the AI-BP patient (lower row)

Figure 2 demonstrates exemplary baseline and follow-up PDFF maps of representative subjects of both treatment groups.

## Discussion

The present study performed BMFF measurements at the lumbar spine of postmenopausal breast cancer patients over a 12-month period using CSE-MRI. In contrast to subjects receiving AI only, the vertebral bone marrow PDFF significantly increased in subjects receiving combined AI and BP therapy.

Vertebral bone marrow water-fat composition has been shown to be significantly altered in osteoporosis and therefore been proposed as advanced imaging biomarker for fracture risk prediction [28, 29]. The loss of trabecular bone in postmenopausal women is primarily caused by estrogen deficiency. Therefore, one important side effect of AI therapy is the promotion of bone loss which can be counteracted by the administration of BP. The present study found that vertebral bone marrow PDFF significantly increased from 45.66 to 48.40% in subjects receiving combined AI and BP therapy based on CSE-MRI measurements. This absolute change of 2.74% is higher than the previously reported reproducibility error of vertebral BMFF measurements of 1.7% [27]. Our findings are at odds with results from previous studies investigating the effect of BP therapy on BMFF quantified by MRS [25] as well as bone biopsies [30]. Those studies reported a significant reduction of BMFF in postmenopausal women receiving BP therapy. However, the two mentioned studies only investigated the effect of BP treatment on bone marrow adiposity in postmenopausal women not receiving any additional medication. In the literature, there is no previous study investigating the effect of BP therapy on postmenopausal women simultaneously receiving AI therapy. Although the detailed physiological and biochemical pathways are still not completely understood it is well established that estrogen is important for maintaining BMD. The protective effects of estrogen on bone health can be explained by several mechanisms. It stimulates osteoclast apoptosis and suppresses osteoblast and osteocyte apoptosis and thereby increases the lifespan of bone building cells and decreases the lifespan of bone resorbing cells. Furthermore, estrogen represses pro-osteoclastic cytokines, such as TNFα, IL-1, IL-6 and IL-7 and down-regulates osteoclastogenesis via the RANKL pathway [31, 32]. It has been shown that the effects of estrogen on bone cells are mediated via estrogen receptors ERα and ER​β. Interestingly, ERα is expressed at a higher level in cortical bone and ERβ is expressed at a higher level in trabecular bone [32]. Estrogen has also been shown to have effects on adipogenesis through regulation of adipocyte precursor proliferation and expression of adipocyte differentiation factors [33–36].

It becomes clear that by reducing estrogen synthesis AI therapy decreases BMD resulting in impaired bone health. BP have been shown to enhance osteoclast apoptosis and thus decrease their lifespan [37, 38] as well as increase osteoblast lifespan [39]. Thus, the cellular and molecular pathways through which AI and BP affect bone turnover as well as bone marrow composition overlap to some extent, but are not completely identical. This could be a potential explanation why BP therapy has a different effect on patients receiving AI therapy than on patients not receiving any estrogen suppressing therapy.

We observed a significant longitudinal difference in vertebral bone marrow PDFF without corresponding changes in BMD. This could be attributed to the fact that vertebral bone marrow fat content not only depends on osseous changes, but also changes in bone marrow composition itself. A second concomitant explanation might be that bone marrow changes start to occur earlier than bone mass related changes. Our findings suggest a higher sensitivity of PDFF measurements to medication induced changes than DXA-based BMD. The fact that there was a close to significant BMD increase in the patient group receiving AI and BP therapy potentially implicates that the effect of BP on BMD is stronger than on PDFF. This can be considered additional evidence that osteoporosis related BMFF changes are not exclusively a result of the replacement of bone by adipose tissue.

The present study is not without limitations. There was no group of age-matched healthy controls. Since aging itself is a contributing factor for bone loss and increased bone marrow adiposity, in particular in postmenopausal women, such a control group would be beneficial in order to better assess the effects of AI und BP therapy. However, in [40] mean and standard deviation of lumbar VBM PDFF values of healthy female subjects of different age groups were analyzed. There was an increase in PDFF from 48.8 ± 7.7% to 50.5 ± 8.2% between the sixties and seventies age group, amounting to a relative PDFF increase of 3.5%, over a 10-year period of time. Admittedly, considering this data as a reference is not equivalent to a dedicated age-matched healthy control group. However, it should provide sufficient confidence that, firstly, the baseline PDFF values of the present study are within the range of healthy controls and, secondly, the 12-month longitudinal effects on marrow adiposity observed in the present study are not only the result of aging.

Another limitation of the present study is the relatively small sample size as well as the relatively short observation period. Performing future follow-up measurements will improve the assessment of treatment associated changes over a longer period of time. This could help to better characterize effects of combined AI and BP therapy on vertebral PDFF and reveal longer-term longitudinal effects.

## Conclusions

Over a 12-month period vertebral PDFF assessed with CSE-MRI significantly increased in subjects receiving combined AI and BP therapy. The present results are not in line with previous results regarding the effect of only BP therapy on BMFF. However, there is no previous study on the combined effect of AI and BP therapy on BMFF. Performing additional follow-up measurements to assess longitudinal effects over a longer time period might help to further characterize the longer-term effects of combined AI and BP therapy on vertebral PDFF.

## Data Availability

The datasets used and/or analyzed during the current study are available from the corresponding author on reasonable request.

## Abbreviations

∆rel _BMD_: Relative longitudinal change in BMD
∆rel _PDFF_: Relative longitudinal change in PDFF
∆rel: Relative longitudinal change
A/P: Anterior/posterior
AI: Aromatase inhibitor
BMD: Bone mineral density
BMD_baseline_: Bone mineral density at baseline measurement
BMD_follow-up_: Bone mineral density at follow-up measurement
BMFF: Bone marrow fat fraction
BMI: Body mass index
BP: Bisphosphonates
CSE: Chemical shift encoding
CSE-MRI: Chemical shift encoding-based water-fat MRI
DXA: Dual energy X-ray absorptiometry
FOV: Field of view
MITK: Medical Imaging Interaction Toolkit
MRI: Magnetic resonance imaging
MRS: Magnetic resonance spectroscopy
PDFF: Proton density fat fraction
PDFF_baseline_: Proton density fat fraction at baseline measurement
PDFF_follow-up_: Proton density fat fraction at follow-up measurement
ROI: Region of interest
T: Tesla
TE: Echo time
TR: Time of repetition
VBM: Vertebral bone marrow
